# Ultra-high-throughput DArTseq-based silicoDArT and SNP markers for genomic studies in macadamia

**DOI:** 10.1371/journal.pone.0203465

**Published:** 2018-08-31

**Authors:** Mobashwer Alam, Jodi Neal, Katie O’Connor, Andrzej Kilian, Bruce Topp

**Affiliations:** 1 Centre for Horticultural Science, Queensland Alliance for Agriculture and Food Innovation, the University of Queensland, Nambour, Queensland, Australia; 2 Department of Agriculture and Forestry, Maroochy Research Facility, Nambour, Queensland, Australia; 3 Diversity Arrays Technology Pty Ltd, University of Canberra, Monana St., Canberra ACT, Australia; Washington State University, UNITED STATES

## Abstract

Macadamia (*Macadamia integrifolia*, *M*. *tetraphylla* and hybrids) is an Australian native nut crop and has a significant economic value in the food industries worldwide. Long juvenility along with traditional breeding strategies impede quick genetic improvement of this crop. The existing cultivars constitute only second to fourth generation of the wild germplasm in the rainforest. The utilisation of molecular markers for genomic selection and genome-wide association studies may accelerate genetic gains. Identification of a robust, reproducible, and cost-effective marker system is instrumental in increasing the efficiency of genomic studies. This study is the first to report the potential of two ultra-high-throughput diversity array technology (DArT) markers (silicoDArT and SNP) in macadamia. Both markers were used to identify the genetic diversity and population structure in 80 macadamia cultivars. Parentage analysis of 25 scions in a rootstock trial was conducted to confirm plant identity where recorded identities did not corroborate with phenotypic field observations. A total of 22,280 silicoDArT and 7,332 SNP markers were reported, of which 11,526 silicoDArT and 3,956 SNP markers were used for analyses after screening with quality control parameters including >95% call rate, >95% reproducibility, and >0.05 one ratio. The average polymorphic information content (PIC) values of silicoDArT and SNP markers were 0.29 and 0.21, respectively. Genetic variance among the cultivars ranged from 0.003 to 0.738 in silicoDArT and 0.004 to 0.412 in SNP markers. Four distinct population groups were identified from SNP data analysis. Most of the accessions used in this study were descended from two or more populations. Cluster analysis clearly separated genotypes of distinct origins, such as the Hawaii Agricultural Experiment Station and Hidden Valley Plantation accessions. Two wild accessions of *Macadamia jansenii* and *M*. *ternifolia* were found to be distantly related to the cultivars. Wild germplasm individuals and their hybrids with cv. ‘660’ formed separate clusters, suggesting that crossing between wild and cultivated genepools can extend genetic diversity. DArTseq-based SNP markers were successfully utilized to confirm the genetic identity of 25 scions in a rootstock trial. Our study suggests that DArT platforms are a robust system for the facilitation of genomic studies with regard to macadamia.

## Introduction

Macadamia is a cross-pollinated tree and constitutes a nut crop industry with a high value kernel [1, 2]. Its unique flavours, multipurpose uses, and long shelf life have increased the demand for macadamia nuts worldwide. The genetic improvement of Australian macadamia cultivars is still at an early stage. Though the crop was first domesticated one and a half centuries ago [1, 3, 4], existing cultivars are only second to fourth generations from their wild progenitors [4, 5]. Until recently, worldwide macadamia improvement programs were mostly dependent on pedigree analysis and phenotypic characterization [6–9], subjected to the inaccuracy involved in the selection of elite accessions due to the effects of environment and genotype-environment interactions. To reduce inaccuracies in selection procedure and to accelerate breeding efficiency, genomic techniques may be used as a tool of macadamia breeding program [10].

Molecular markers have been utilized in several crop species to ascertain the genetic diversity in the gene pool [11], identify QTLs and candidate genes conferring valuable traits [12, 13], authenticate plant identity and the parentage of hybrids [14], generate data for gene expression profiling [15], and predict the genetic potentiality/performance of individual cultivars [16]. Over the last few decades, several marker technologies have been developed for macadamia, primarily for the study of genetic diversity. Some of the important markers used in macadamia are isozyme [17], randomly amplified DNA fingerprinting (RAF) [18, 19], random amplified polymorphic DNA (RAPD) [20, 21], amplified fragment length polymorphism (AFLP) [22, 23], randomly amplified microsatellite fingerprinting (RAMiFi) [24] and microsatellite [25–27] markers. Limitations associated with these marker systems include low marker density, poor genome coverage, and less cost-effectiveness. In this perspective, sequence-based single nucleotide polymorphism (SNP) markers developed through automated sequencers can constitute an important choice for molecular studies due to their wide and uniform genome coverage with high-throughput and cost-effectiveness [28]. There is an increasing demand for the development of ultra-high-throughput low cost assays to facilitate the genotyping of individuals with the use of a large number of high-density markers that cover the entire genome. High-density array-based oligonucleotide markers such as single feature polymorphisms (SFPs), restriction site-associated DNA (RAD), and diversity array technology (DArT) provide a way to achieve this goal of development of cost-efficient ultra-high-throughput genotyping systems.

In 2001 [29], Diversity Array Technology Pty Ltd (DArT, Canberra, ACT, Australia) developed cost-effective sequence-independent ultra-high-throughput marker systems. DArT develops markers through a microarray hybridization method and can produce thousands of polymorphic loci in a single assay. This marker platform has been used for whole-genome scanning of a range of crop species [30–36]. Over the last decade, DArT has generated two types of markers: i) silicoDArT and ii) SNP markers. SilicoDArT markers are microarray markers that are dominant and scored for the presence or absence of a single allele. DArTseq-based SNPs are co-dominant markers. Both types of markers have been successfully applied in several crop species for genetic diversity [37–41], genetic mapping [36, 42–44], and population structure [45, 46] studies. The present study is the first to utilize the DArT platforms in macadamia; we present the development of DArT marker platforms, and compare and analyse the usefulness of silicoDArT and SNP markers for genomic studies. DArT markers were applied to investigate the genetic diversity in cultivated and wild germplasm. DArT markers were also tested for utilization in plant identification.

## Materials and methods

### Plant materials

Macadamia cultivars of multiple origins including from Hawaii Agricultural Experiment Station (HAES), Hidden Valley Plantation (HVP), Israeli and Australian selections, accessions from wild germplasm, and progeny from the Australian industry macadamia breeding program (Australian elite selections, dwarves, breeding progeny and hybrids of wild germplasm) were employed for genetic diversity and population structure analysis (Table 1). Leaf samples from seven cultivars were collected from each of two different plants to use as biological replicates. All 21 cultivars were genotyped twice to use as technical replicates. Twenty-five scions from a rootstock trial at Bundaberg, Queensland, were also tested for plant identity to solve a mismatch with phenotypic characteristics observed in the field.

**Table 1:** List of macadamia cultivars used in genetic diversity study. HAES Hawaiian Agricultural Experiment Station; HVP Hidden Valley Plantation.

Accessions	Origin	Female	Male	Reason of study
333	HAES	Unknown	Unknown	Cultivar
344	HAES	Unknown	Unknown	Cultivar
660	HAES	Unknown	Unknown	Cultivar
741	HAES	Unknown	Unknown	Cultivar
762	HAES	Unknown	Unknown	Cultivar; Technical replicate
781	HAES	Unknown	Unknown	Cultivar
791	HAES	Unknown	Unknown	Cultivar
804	HAES	Unknown	Unknown	Cultivar
814	HAES	Unknown	Unknown	Cultivar
816	HAES	Unknown	Unknown	Cultivar
842	HAES	Unknown	Unknown	Cultivar
849	HAES	Unknown	Unknown	Cultivar
842/H2	HAES/Australian cultivar	Unknown	Unknown	Technical replicate
A16	HVP	Release	Unknown	Cultivar
A203	HVP	344	Unknown	Cultivar
A268	HVP	344	Unknown	Cultivar
A4	HVP	Release	Unknown	Cultivar; Technical replicate
AM-4-8	Australian industry breeding	705	A16	Technical replicate
AM-9-28	Australian industry breeding	660	NG18	Technical replicate
Beaumont	Australian cultivar	Unknown	Unknown	Cultivar
D4	Australian cultivar	Unknown	Unknown	Cultivar
Daddow	Australian cultivar	Unknown	Unknown	Cultivar; Technical replicate
DW1	Australian industry breeding	NG8	762	Dwarf
DW2	Australian industry breeding	NG8	762	Dwarf
DW3	Australian industry breeding	Yonik	NG8	Dwarf
DW4	Australian industry breeding	Yonik	NG8	Dwarf
H2	Australian cultivar	Unknown	Unknown	Cultivar
IMCDW	Australian industry breeding	Unknown	Unknown	Dwarf
Mjan	Wild germplasm	Unknown	Unknown	Wild *M*. *jansenii*
Mtern	Wild germplasm	Unknown	Unknown	Wild *M*. *ternifolia*
NG18	Australian cultivar	Unknown	Unknown	Cultivar
NG29	Australian cultivar	Unknown	Unknown	Cultivar
NG8	Australian cultivar	Unknown	Unknown	Cultivar
Own Venture	Australian cultivar	Unknown	Unknown	Cultivar
QB-10-111Q	Australian industry breeding	246	A16	Australian elite
QB-10-93J	Australian industry breeding	A16	781	Australian elite
QB-11-14	Australian industry breeding	660	NG18	Technical replicate, Breeding progeny
QB-11-35T	Australian industry breeding	849	Daddow	Australian elite
QB-11-80C	Australian industry breeding	814	A16	Australian elite
QB-13-115R	Australian industry breeding	842	Daddow	Australian elite
QB-14-25P	Australian industry breeding	A16	814	Australian elite
QB-14-93G	Australian industry breeding	Daddow	246	Australian elite
QB-15-14	Australian industry breeding	660	NG18	Technical replicate, Breeding progeny
QB-15-37M	Australian industry breeding	Daddow	A16	Australian elite
QB-16-41H	Australian industry breeding	Daddow	A16	Australian elite
QB-19-14	Australian industry breeding	705	A16	Technical replicate, Breeding progeny
QB-2-14	Australian industry breeding	NG8	762	Technical replicate, Breeding progeny
QB-2-46E	Australian industry breeding	246	A16	Australian elite
QB-26-3	Australian industry breeding	660	NG18	Technical replicate, Breeding progeny
QB-31-5	Australian industry breeding	A4	781	Technical replicate, Breeding progeny
QB-35-10	Australian industry breeding	Yonik	NG8	Technical replicate, Breeding progeny
QB-36-3	Australian industry breeding	741	Daddow	Technical replicate, Breeding progeny
QB-5-7	Australian industry breeding	A4	781	Technical replicate, Breeding progeny
QB-5-81	Australian industry breeding	NG8	762	Technical replicate, Breeding progeny
QB-6-16S	Australian industry breeding	A16	814	Australian elite
QB-6-17	Australian industry breeding	Own Venture	NG7	Technical replicate, Breeding progeny
QB-6-71	Australian industry breeding	816	842	Breeding progeny
QB-6-73N	Australian industry breeding	842	A16	Australian elite
QB-6-79I	Australian industry breeding	A16	814	Australian elite
QB-7-109L	Australian industry breeding	842	Daddow	Australian elite
QB-7-11	Australian industry breeding	NG8	762	Technical replicate, Breeding progeny
QB-7-5	Australian industry breeding	NG18	695	Technical replicate, Breeding progeny
QB-7-74O	Australian industry breeding	Daddow	A4	Australian elite
QB-8-87F	Australian industry breeding	816	A4	Australian elite
QB-9-5	Australian industry breeding	A4	781	Technical replicate, Breeding progeny
QB-9-72K	Australian industry breeding	842	Daddow	Australian elite
RQB-8-3-22	Australian industry breeding	741	Unknown	Technical replicate, Breeding progeny
TF-11-1	Australian industry breeding	660	*Wild M*. *jansenii*	Hybrid of wild *M*. *jansenii*
TF-15-1	Australian industry breeding	660	Unknown	Breeding progeny
TF-23-4	Australian industry breeding	660	*Wild M*. *ternifolia*	Hybrid of wild *M*. *ternifolia*,
TF-23-9	Australian industry breeding	660	*Wild M*. *jansenii*	Hybrid of wild *M*. *jansenii*
TF-34-11	Australian industry breeding	660	*Wild M*. *jansenii*	Hybrid of wild *M*. *jansenii*
TF-37-1	Australian industry breeding	660	*Wild M*. *ternifolia*	Hybrid of wild *M*. *ternifolia*
TF-43-23A	Australian industry breeding	A16	781	Australian elite
TF-44-15D	Australian industry breeding	Daddow	246	Australian elite
TF-47-16	Australian industry breeding	660	Unknown	Breeding progeny
TF-48-8	Australian industry breeding	660	Unknown	Breeding progeny
TF-9-20	Australian industry breeding	660	Unknown	Breeding progeny
TF-9-22B	Australian industry breeding	849	Daddow	Australian elite
Yonik	Selection from Israel	Unknown	Unknown	Cultivar

### DNA extraction

Samples obtained from newly flushed young leaves were collected from arboretums and progeny trials of the Australian industry macadamia breeding program [47] in 2013 and 2014 growing seasons. Leaf samples were sealed in a zip-lock bag labelled with the corresponding tree barcode, kept cool in insulated bags with freezer blocks and sent to DArT (next day) for DNA extraction. Total genomic DNA was extracted by adhering to the modified CTAB protocol [48, 49] as described by Kilian et al., (2012) [50]. The quality and quantity of the DNA samples were evaluated through a spectrophotometric analysis in DS-11FX series spectrophotometer/fluorometer (Denovix, Wilmington, DE, USA), followed by running agarose gel electrophoresis (1.2% agarose). The DNA concentration was adjusted within the range of 50–100 ηg μl^–1^.

### Genotyping macadamia accessions using silicoDArT and SNP markers

The high-throughput DArTseq technology was used to genotype macadamia cultivars. In this technology, the PstI-based complexity reduction method [39] was applied for the enrichment of genomic representation with single copy sequences. This method involved the digestion of DNA samples with a rare cutting enzyme PstI, paired with a set of secondary frequently cutting restriction endonucleases (RE), ligation with site-specific adapters, and amplification of adapter-ligated fragments. The secondary frequently cutting RE enzymes employed in this study were *Alu*I, *Apo*I, *Ban*II, *Bsp*1286I, *Bst*NI, *Hae*III, *Mse*I, *Rsa*I, *Msp*I, *Hpa*II, *Mse*I, *Taq*I, and *Hha*I. Post digestion with a PstI-RE pair, a PstI overhang compatible oligocleotide adapter (5′-CAC GAT GGA TCC AGT GCA-3′ annealed with 5′-CTG GAT CCA TCG TGC A-3′) was ligated, and the adapter-ligated fragments were amplified in adherence to the prescribed standard procedures [39]. To develop SNP and silicoDArT markers, the DArTseq technology was optimized using two PstI-compatible adapters corresponding to two different RE overhangs. The genomic representations were generated following the procedures described by Kilian et al. [50] and PstI+HhaI was selected as the most appropriate complexity reduction method. Next-generation sequencing technology was implemented using HiSeq2000 (Illumina, USA) to detect SNPs and silicoDArT markers. The sequence data was analyzed using DarTsoft14, an automated genotypic data analysis program and DArTdb, a laboratory management system. Markers were scored ‘1’ for presence, and ‘0’ for absence and ‘-’ for failure to score. Two technical replicates of the DNA samples of each of 21 cultivars were genotyped to calculate the reproducibility of the marker data.

### Quality analysis of marker data

The markers were tested for reproducibility (%), call rate (%), polymorphism information content (PIC) and one ratio. Scoring of reproducibility involved the proportion of technical replicate assay pairs for which the marker score exhibited consistency. The call rate determined the success of reading the marker sequence across the samples and was estimated from the percentage of samples for which the score was either ‘0’ or ‘1’. PIC is the degree of diversity of the marker in the population and showed the usefulness of the marker for linkage analysis. One ratio constitutes the proportion of the samples for which genotype scores equalled ‘1’.

### Genetic dissimilarity analysis

Genetic dissimilarity matrices were constructed in DARwin v. 6.0.13 [51] to identify the genetic relationships among the cultivars. Weighted neighbour-joining dendrograms were constructed in both marker platforms. Clade strength in the dendrograms was tested by 20,000 bootstrap analyses. Biological and technical replicates of 28 accessions were compared to identify the reliability of the markers using the dissimilarity index between the replicates of each accession.

### Population structure and genetic diversity analysis

The genetic structure of the germplasm was analyzed using STRUCTURE v.2.3.4 [52] and GenAlEx v.6.5 [53]. The number of hypothetical subpopulations (K) was estimated with the STRUCTURE software through the application of a Bayesian clustering approach for the organisation of genetically similar cultivars into the same subgroups. Ten individual Markov Chain Monte Carlo (MCMC) simulations were conducted for each K-value from 1 to 10 with a burn-in length of 50,000, followed by 100,000 iterations. The admixture model was applied without using any prior population information. The log-likelihood of the observed data for each K-value was calculated and compared across the range of K values. The best K-value was estimated based on the membership coefficient (Q) for each individual in each cluster. The Q values indicate the level of relatedness of each accession to various subgroups. The STRUCTURE results were subsequently analyzed by the STRUCTURE HARVESTER application [54] (http://taylor0.biology.ucla.edu/structureHarvester/) to identify a distinct peak in the change of likelihood (ΔK) at the true value of K. GenAlEx was used to perform Principal Coordinates Analysis (PCoA), based on the standardized covariance of genetic distances calculated for the markers under evaluation, using 999 permutations. PCoA explains the genetic distances among the accessions.

### Determining plant identity

In a rootstock trial at Bundaberg we identified that the phenotypic characteristics of 25 scions did not corroborate with their recorded variety. Using SNP markers we conducted PCoA and parentage analysis of all the mismatched scions and the results were compared with phenotypic variety allocations.

To determine plant identity, initially allelic variations were observed between the possible varieties and the mismatched cultivars using PCoA in GenAlEx. Later, the analysis of plant identity was performed with Cervus v.3.0.7 [55] to detect and confirm the candidate plant ID. In cases where the plant ID remained indeterminate, parentage analysis was conducted, allowing 40 simulated parents and 10,000 simulated offspring. The natural logarithm of the likelihood ratio, logarithm of odds (LOD), scores were provided for each candidate parent and the offspring-parents trio along with the confidence of these scores at strict (95%) and relaxed (80%) levels. Based on the analysis, the parent with the highest LOD is denoted as the putative parent. A positive LOD score indicates that the parent is more likely to be the true parent in comparison to one drawn at random from the population; negative LOD scores indicate that the parent is less likely to be the parent than one drawn at random from the population [55].

## Results

### Marker quality analysis

**Fig 1:**
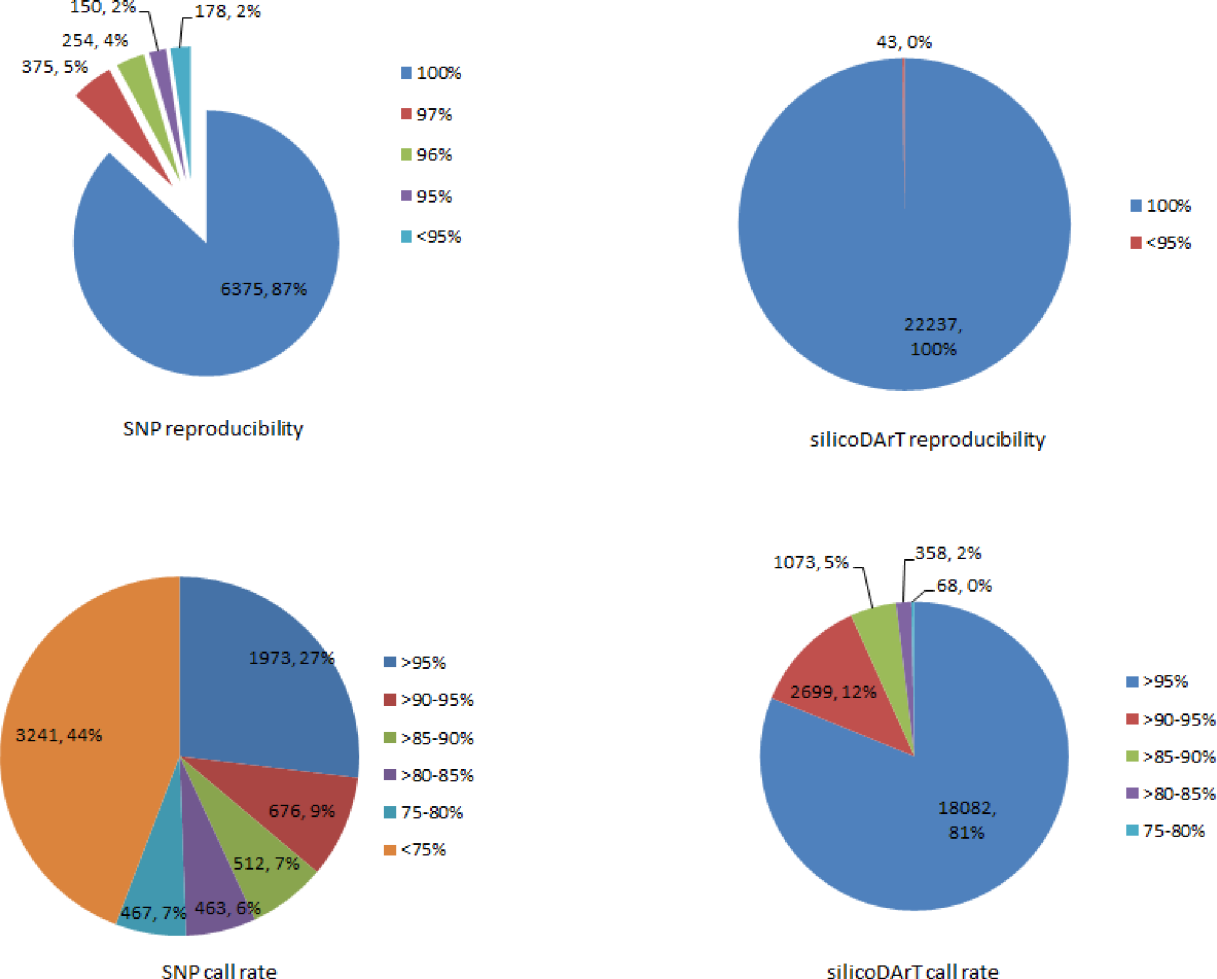
Distribution of silicoDArT and SNP marker data for several quality parameters.

**Fig 2:**
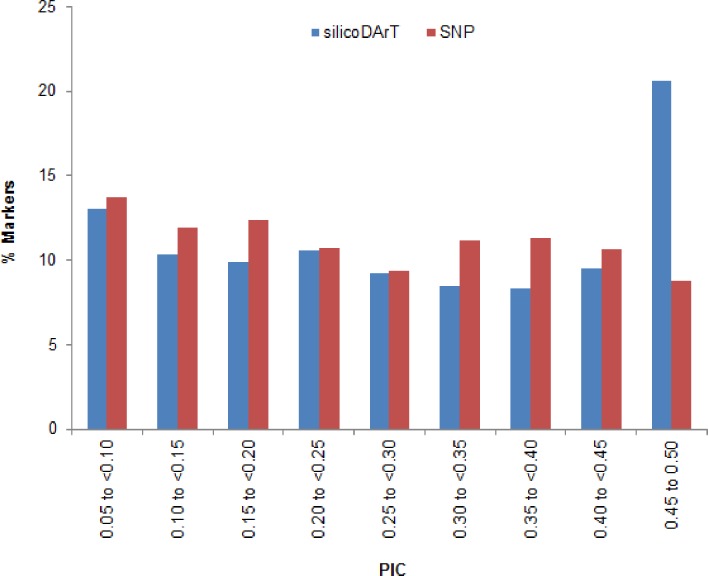
Distribution of PIC values of silicoDArT and SNP markers used for genomic studies in macadamia.

Through the application of the complexity reduction method, a total of 22,280 polymorphic silicoDArT (S1 Table) markers were generated, of which 353 were aligned with the marker sequence obtained from bacteria (NCBI) and 154 from expressed sequenced tags (EST) of several plant species. We report these DArT markers for the first time, though the chromosomal location is yet to be described. Most of the markers (22,237) showed ≥95% reproducibility. All the identified silicoDArT markers had a call rate value ≥75% (Fig 1) with an average value of 97.44 (S1 Table). However, low frequency markers can affect the statistical analysis [56]. As such, 10,711 markers with extremely low one ratio (<0.05) were not considered in the analysis. In total, 11,526 silicoDArT markers cleared all the quality parameters and were selected for the study. Among the 11,526 informative markers, around 21% were observed in PIC class 0.45 to 0.50 and 13% in 0.05 to 0.10 class (Fig 2). PIC values of the remaining markers were distributed almost equally (8–10%) across the rest of the marker groups. Therefore, the median (0.28) was located extremely close to the average PIC value of 0.29 (S3 Table), and the data exhibited approximately equitable distribution on either side of the median.

A total of 7,332 SNP markers (S2 Table) were identified, and had an average of 99% reproducibility and 76% call rate. Around 98% SNP markers had ≥95% reproducibility, of which 6,375 were found to be 100% reproducible (Fig 1). The call rate exhibited variance ranging from 38% to 100%. Around 44% of SNP markers displayed a <75% call rate (Fig 1), and were therefore not considered for this study. For the remaining markers, 2,650 showed a >90% call rate. All the identified markers had >0.5 average one ratio. Considering all the quality parameters, 3,956 SNP markers were used for subsequent analysis. These markers were determined to be highly informative with an average PIC value of 0.21, and 0.19 median (S3 Table). Around 14% of markers were in the lowest PIC value range (0–0.05) and 9% in the highest PIC value range (0.45 to 0.50) (Fig 2). The remaining PIC value groups exhibited an approximately similar marker frequency value ranging from 10 to 12% each.

### Genetic relationships among cultivars

**Fig 3:**
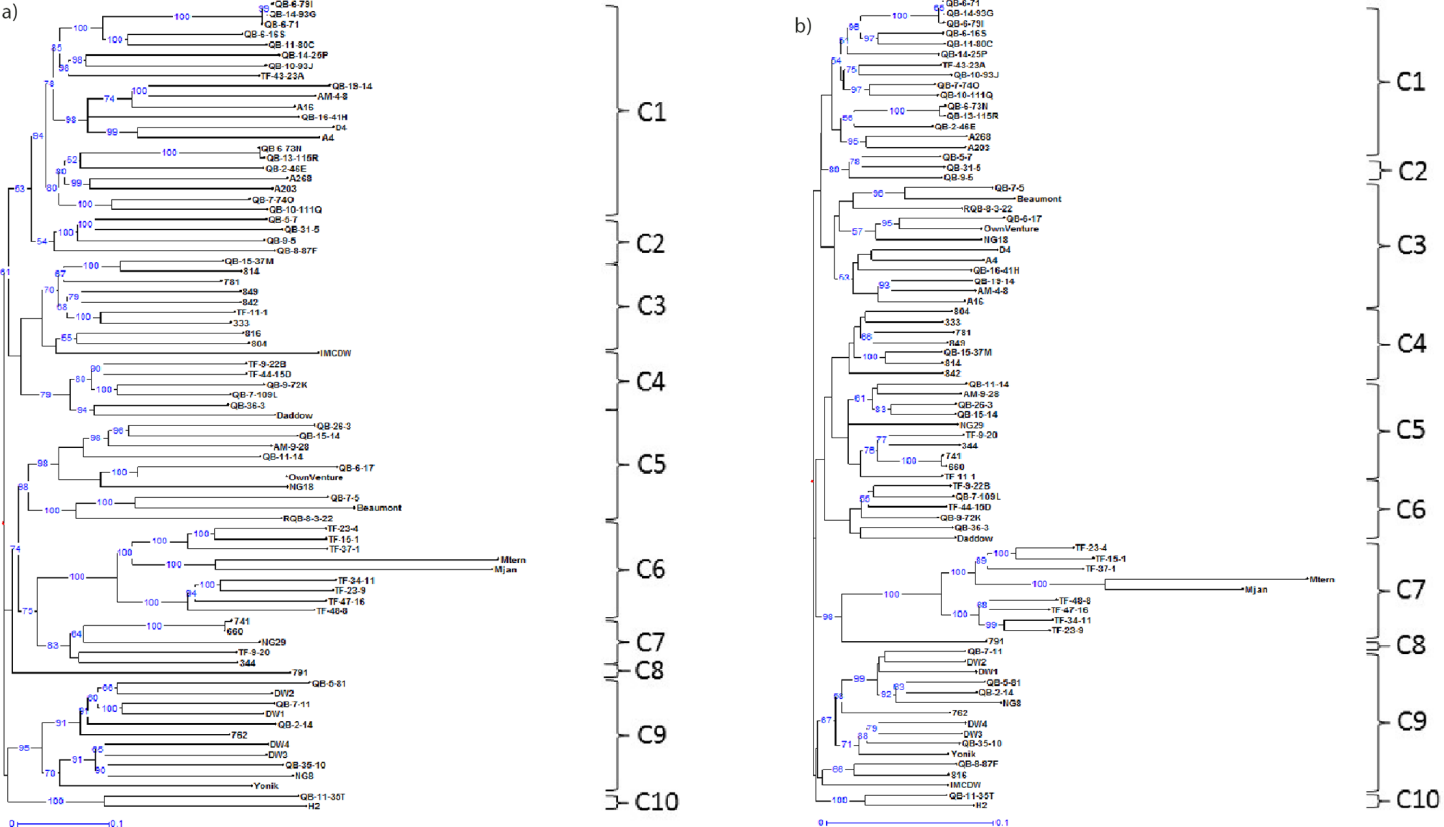
Genetic relationships among 80 macadamia accessions, and 7 biological and 21 technical replicates. (a) Weighted neighbour-joining dendrogram based on silicoDArT markers. (b) Weighted neighbour-joining dendrogram based on SNP markers.

The genetic dissimilarities among the cultivars estimated through the silicoDArT markers ranged from 0.003 to 0.738 (S4 Table). The technical and biological replicates of the cultivars revealed the least amount of genetic dissimilarity (Fig 3), ranging from 0.004 to 0.02. Among the cultivars, ‘660’ displayed exceptionally close genetic similarity with ‘741’ (dissimilarity indexes: 0.004 to 0.008). This value was similar to that of the biological replicates of ‘741’. The dissimilarity indices of *M*. *jansenii* and *M*. *ternifoli*a with the existing cultivars ranged from 0.590 to 0.738, suggesting that the two wild accessions are distantly related to the cultivated gene pool. The Australian industry breeding lines (TF-11-1, TF-23-4, TF-23-9, TF-34-11 and TF-37-1) developed through the hybridization of wild germplasm with commercial cultivars further exhibited wide genetic distance from Australian macadamia cultivars.

The dendrogram obtained with silicoDArT markers produced several small clusters of related cultivars, and most of them contained cultivars that shared parental lines or a similar origin (Fig 3A). We identified nine major groups of clusters. Most of the HVP cultivars clustered in C1, while HAES cultivars were grouped in four other separate clusters. Cultivars ‘816’, ‘804’, ‘814’, ‘781’, ‘849’, ‘842’, and ‘333’ were grouped in C3. In C9, HAES cultivar ‘762’ clustered with ‘NG8’. Other three HAES cultivars (‘741’, ‘660’ and ‘344’) and an Australian selection, ‘NG29’, were grouped in cluster C7. HAES cultivar ‘791’ constituted the sole representative of C8. Wild *M*. *jansenii* and *M*. *ternifolia* and their hybrids (‘TF-23-4, ‘TF-15-1’, ‘TF-37-1’, ‘TF-34-11’, ‘TF-23-9’, ‘TF-47-16,’ and ‘TF-48-8’) formed a separate cluster (C6). Australian elites and selections were distributed across the clusters, which illustrates the wide range of diversity created by the Australian breeding program. The Australian selection, ‘D4’, grouped with HVP cultivars in C1. ‘Beaumont’, ‘NG18’, and ‘Own Venture’ grouped together in C5. Cultivars ‘H2’, and ‘Daddow’ formed two separate clusters, C10 and C4, respectively. We further identified that four dwarf cultivars (‘DW1’, ‘DW2’, ‘DW3’, and ‘DW4’) clustered in the same group (C9) as their common parent ‘NG8’.

SNP markers were also useful for the identification of genetic relationships among Australian cultivars. The range of genetic dissimilarities identified through SNP markers was narrower than that observed through silicoDArT markers. Among the 80 cultivars and 28 replicates, dissimilarity ranged from 0.004 to 0.412 (S5 Table). The genetic dissimilarity index between ‘741’ and ‘660’ (0.013) fell within the range of dissimilarities between technical and biological replicates (0.004 to 0.03).

Similar to silicoDArT markers, SNP markers also developed several clusters of macadamia cultivars based on their relatedness (Fig 3B). Cultivars with common origin or those that shared common parents were clustered together. HAES and HVP cultivars formed distinctly separate clusters. HAES cultivars were grouped in C4, C5, C8 and C9; whereas HVP cultivars were clustered in C1 and C3. As observed with silicoDArT markers, the wild accessions of *M*. *ternifolia* and *M*. *jansenii* were found to be distantly related with other cultivars, and grouped in C7 with their respective hybrids (Fig 3B). We also observed three separate clusters of ‘H2’ (C10), ‘791’ (C8), and ‘Daddow’ (C6) in the SNP analysis. Same as in silicoDArTs, four dwarf cultivars (DW1, DW2, DW3 and DW4) were clustered together (in C9) with their common parent ‘NG8’.

### Population structure and genetic diversity analysis

**Fig 4:**
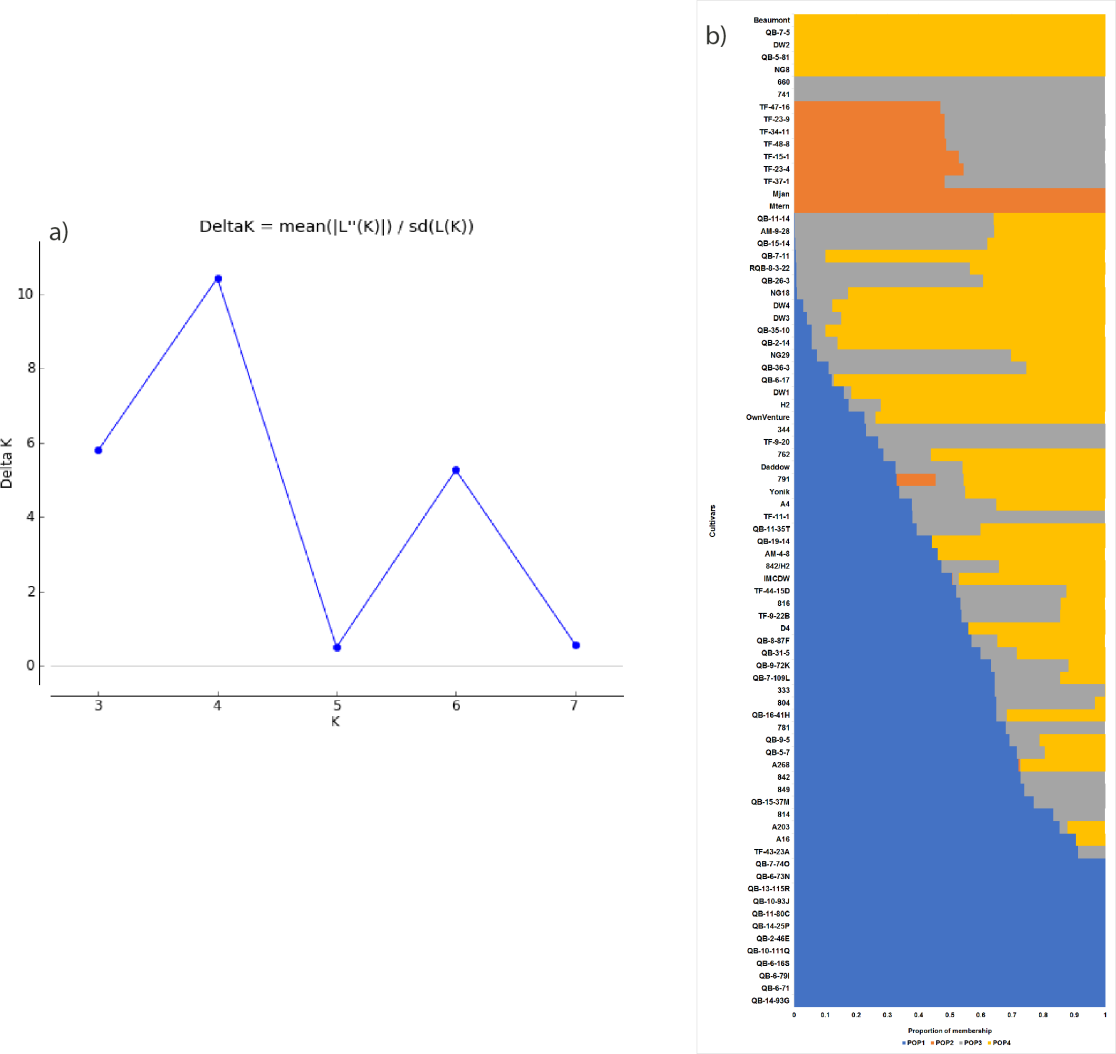
Population structure of 80 macadamia accessions using SNP marker data, as estimated using the model-based Bayesian algorithm implemented in the STRUCTURE program. a) estimation of number of groups (K), b) proportion of assignment of individuals to four population groups.

The model-based Bayesian cluster analysis in STRUCTURE visualized the genetic structure of the population under examination (Fig 4). The values of ΔK, which were estimated from SNP markers peaked at K = 4 (Fig 4A); hence, four distinct groups were found to contribute significant genetic information across cultivars. The sub-populations were denoted as POP1, POP2, POP3 and POP4, and each sub-population contained 41%, 7%, 23%, and 29% of total accessions, respectively (Table 2).

**Table 2:** Genetic divergence among (net nucleotide distance) and within (expected heterozygosity) populations, and the proportion of membership of the population samples.

Population	Net nucleotide distance			Expected heterozygosity	Proportion of membership
	POP2	POP3	POP4		
POP1	0.19	0.08	0.05	0.19	0.41
POP2		0.18	0.18	0.14	0.07
POP3			0.09	0.12	0.23
POP4				0.22	0.29

The genetic diversity within each population was explained through the estimation of the expected heterozygosity, which varied from 0.12 (POP3) to 0.22 (POP4). The expected heterozygosity of POP1 was 0.19 and that of POP2 was 0.14. The genetic divergence among the populations revealed by Nei’s net nucleotide distance (D) indicated that POP2 was widely related to POP1 (D = 0.19), POP3 (D = 0.18), and POP4 (D = 0.18), respectively. The genetic distance observed between POP1 and POP4 (D = 0.05) was the least among the pairs of populations examined (Table 2).

The proportion of membership of individual cultivars in each population is illustrated in the bar plot of the population assignment test in structure analysis (Fig 4B). The estimated proportion of membership (Q) suggested that 12 Australian elite cultivars (‘QB-14-93G’, ‘QB-6-71’, ‘QB-6-79I’, ‘QB-6-16S’, ‘QB-10-111Q’, ‘QB-2-46E’, ‘QB-14-25P’, ‘QB-11-80C’, ‘QB-10-93J’, ‘QB-13-115R’, ‘QB-6-73N’ and ‘QB-7-74O’) were assigned entirely in POP1. *M*. *jansenii* and *M*. *ternifolia* comprised POP2. Two Hawaiian cultivars ‘741’ and ‘660’ were included in POP3. Australian selections ‘NG8’ and ‘Beaumont’ represented POP4 along with the dwarf progeny ‘DW2’, and Australian elites ‘QB-7-5’ and ‘QB-5-81’. The remaining 60 cultivars showed intermediate and/or highly mixed genetic composition and were hence determined as heterogeneous. The hybrid progeny of ‘660’ X *M*. *jansenii* (‘TF-11-1’, ‘TF-23-9’, and ‘TF-34-11’), and ‘660’ X *M*. *ternifoila* (‘TF-23-4’ and ‘TF-37-1’) were assigned in both POP2 and POP3. Most of the HAES cultivars (‘333’, 344’, ‘781’, ‘804’, ‘814’, ‘816,’ and ‘849’) comprised admixtures of POP1 and POP3. Conversely, all HVP accessions were constituted partly of POP4. Two HAES cultivars ‘791’ and ‘762’ also shared large amounts of genetic information with POP4. Cultivar ‘791’ was the only individual allocated to all four populations, and possessed 33% genetic information from POP1, 13% from POP2, 9% from POP3, and 46% from POP4.

**Fig 5:**
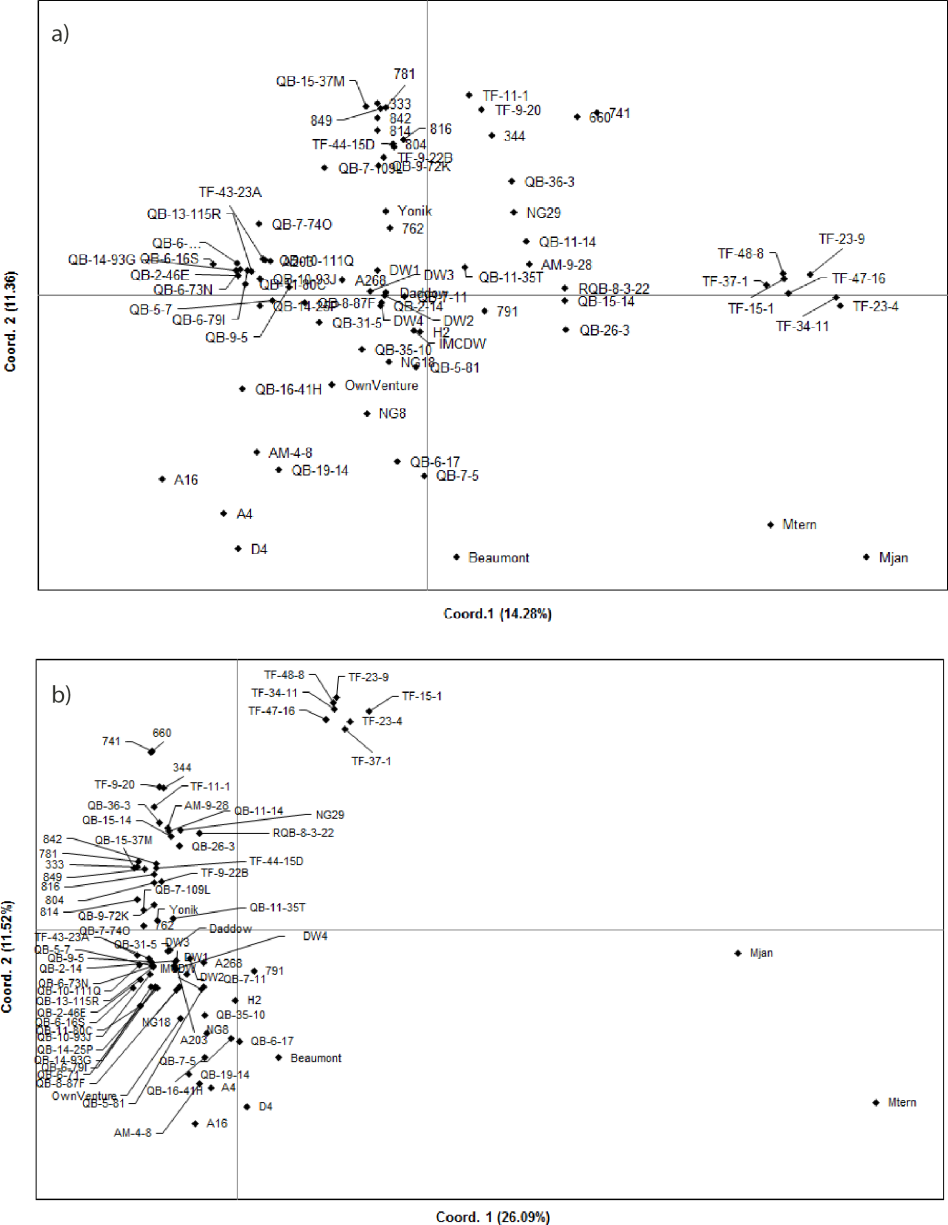
Principal coordinate analysis (PCoA) to explain the genetic diversity across macadamia accessions. a) PCoA based on silicoDArT markers, and b) PCoA based on SNP markers.

PCoA illustrated the genetic divergence among the cultivars (Fig 5). In silicoDArT and SNP markers, the first two axes of the PCoA explained 25.64% and 37.61% of the total genetic divergence, respectively. The population distribution determined by both markers is consistent with the output of population structure analysis (Fig 4B). HAES cultivars were located in the top two quadrants, while HVP and Australian selections were mainly located in the bottom quadrants. Australian breeding lines displayed wide diversity, as they were mostly distributed throughout the three quadrants of the PCoA. Only a few accessions were in the bottom right quadrant of PCoA, which was predominantly occupied by wild germplasm.

### Plant identification using SNP markers

**Fig 6:**
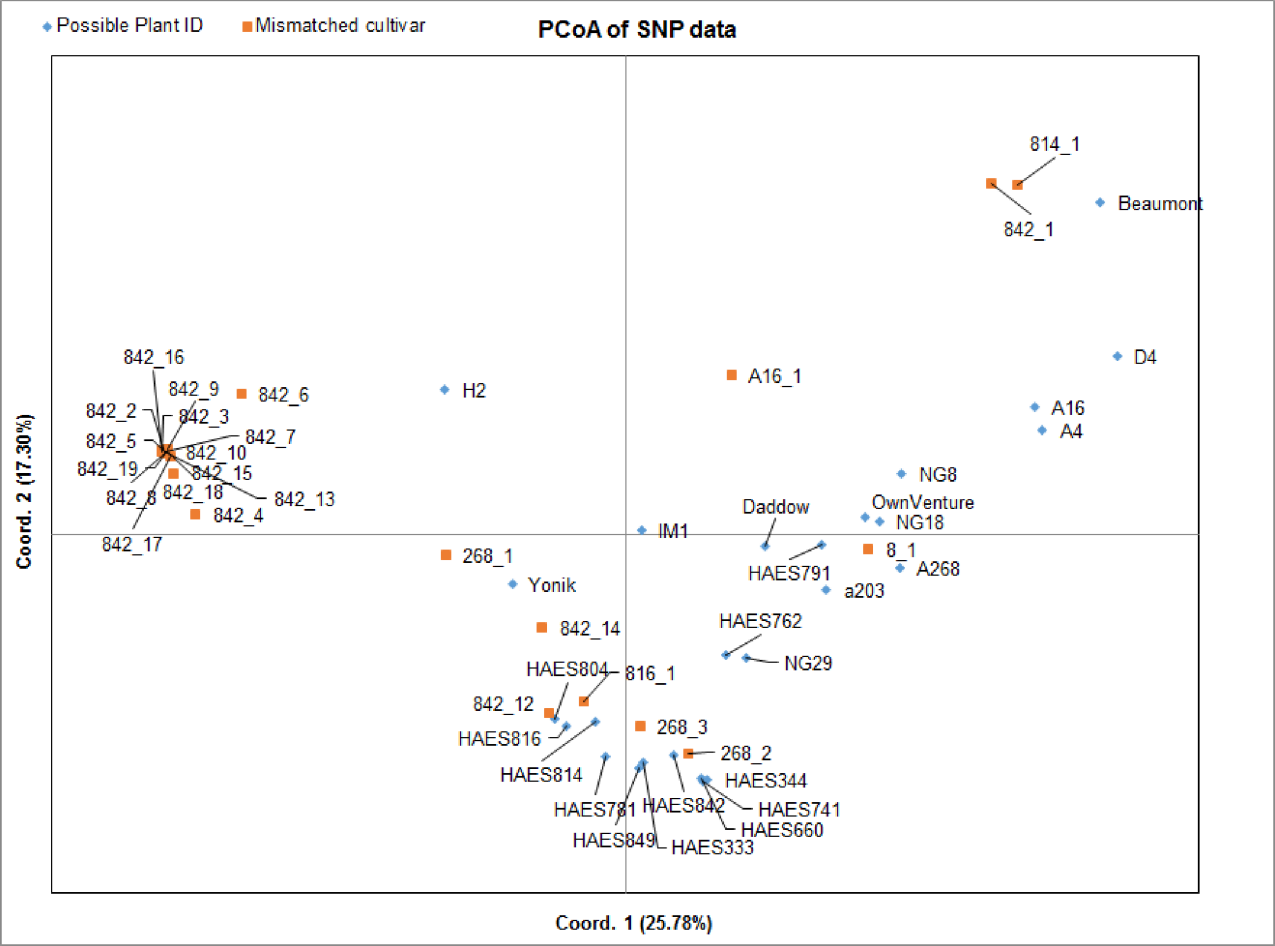
Principal coordinate analysis of SNP markers showing allelic variation among macadamia cultivars and mismatched scions.

PCoA of the SNP markers derived from possible cultivars and mismatched scions illustrated allelic variations among the accessions (Fig 6). Parentage analysis identified that seven scions in the rootstock trial were planted with wrong plant ID and the remaining 18 scions were the seedling progeny of rootstocks ‘Beaumont’ and ‘H2’ (S7 Table).

The first two components of the PCoA explained 43.08% of total variation. Pair wise comparison showed that least allelic variations were observed within the following mismatched scion and cultivar pairs: ‘8_1’ and ‘A268’, ‘268_2’ and ‘344’, ‘268_3’ and ‘842’, ‘814_1’ and ‘Beaumont’, ‘816_1’ and ‘814’, ‘842_1’ and ‘Beaumont’, and ‘842_12’ and ‘816’ (Fig 6 and S6 Table). The parentage analysis (S7 Table) confirmed these seven plant identities and suggested that scions labelled with ‘8_1’, ‘268_2’, ‘814_1’, ‘816_1’ and ‘842_1’ showed a completely congruous match with phenotypic plant IDs (‘A268’, ‘344’, ‘Beaumont’, ‘814’, and ‘Beaumont’, respectively). Plant IDs of ‘268_3’ and ‘842_12’ did not match with phenotypic IDs. Molecular analysis confirmed scion ‘842_12’ as ‘816’, whereas it was phenotypically identified as ‘H2’. Similarly, ‘268_3’ was suggested as ‘814’ in the field, while it was identified as ‘842’ from SNP marker analysis. The candidate mother ID, pair loci mismatching data, and LOD scores suggested that the remaining ‘842’ marked plants have common candidate mother ‘H2’. Trees ‘A16_2’ and ‘268_1’ were indicated to be the progeny of ‘Beaumont’ and ‘H2’, respectively.

## Discussion

### DArT platforms provide cost-effective ultra-high-throughput reliable markers

Our study highlights the suitability of DArT platforms that can be applied for the genomic dissection of macadamia cultivars. A total of 22,237 silicoDArT markers were developed, of which 11,526 markers provided robust information of the macadamia genome in the absence of sequence information. On the other hand, DArTseq SNPs provided 3,956 informative markers. The development of a large number of these high-throughput markers provided an opportunity to anchor the markers on a recently released macadamia reference genome, which is yet to be completed [57].

The quality parameter of both silicoDArT and SNP markers in macadamia were comparable with that of other species. The average PIC values of both markers in macadamia was similar to values identified in DArT markers of sugar beet (0.28) [58] and *Asplenium* fern (0.20) [59], but lower than that of sorghum (0.41) [36], cassava (0.42) [60], and wheat (0.44) [61], and higher than SNP markers in watermelon (0.13) [62] and *Lesquerella* (0.12) [63]. The average PIC values of silicoDArT was greater than that of SNP markers. Around 30% of silicoDArT and 20% of SNP markers showed PIC values within the range of 0.40 to 0.50. The silicoDArT markers were therefore more informative than SNP markers. The abundance of silicoDArT and SNP markers may achieve better genome coverage through the sampling of a greater number of points in the whole genome, as marker density has a high correlation with gene density [50, 64]. Previous studies in macadamia using other platforms utilized only a small number of molecular markers, for example, O’Connor et al. (2015) used only 11 microsatellite markers [25]. Therefore, both silicoDArT and SNP markers may better suit for genetic diversity studies, association/linkage mapping and/or sequence based physical mapping in macadamia. Additionally, the co-dominant inheritance pattern of SNP markers may increase the utility of DArT platforms for genetic identity and parentage analysis.

In comparison with the other existing marker technologies like microsatellite markers, DArT markers are pertinent to high-throughput work and have merits in terms of cost effectiveness and time aspect [65]. The cost of marker development of silicoDArT and SNP markers are the same. Due to the higher number of markers produced in silicoDArT, the average cost per data point is less than SNP markers. However, the effectiveness of both platforms varies depending on the type of application. For genetic diversity and linkage mapping large number of silicoDArT markers are suitable. However, for genetic identity and product quality testing, both markers can perform equally. Due to the opportunity to track alleles from parental genotypes, the co-dominant SNP markers are more suitable in plant identity and parentage analysis than silicoDArT.

### DArT markers successfully evidenced the historical background and pedigree relationships of the cultivars

The genetic diversity analysis illustrated the historical development of macadamia cultivars. Macadamia cultivars have been developed by few breeding programs. The macadamia breeding program in Hawaii began in approximately 1948 and continued to 1990, producing many HAES cultivars which are still dominant in plantings around the world [66]. Australian varieties including ‘Beaumont’, ‘D4’, and ‘Daddow’ are the product of 1952-onwards seedling surveys in Australia, after observation of the Hawaiian industry success. Hidden Valley Plantation (HVP), which commenced in 1972 in Beerwah, Queensland, Australia released successful ‘A’ series cultivars from open-pollinated progeny of HAES and Australian varieties. Genetic diversity analysis clearly separated most of the accessions of these three groups. Accessions of Australian industry breeding efforts were selected from the crossing program initiated in 1992 and included HAES, HVP and Australian cultivars in the parentage, hence are scattered across all clusters.

The statistical analysis of DArT data sets showed highly consistent results obtained for genetic diversity, population structure and PCoA. The pedigree relationships obtained through genetic diversity and population structure analysis are in line with the results of previous studies [17, 18, 23]. Two HAES cultivars ‘660’ and ‘741’, which were selected from same orchard at Glaisyer (at Hawaii) showed strong genetic similarity, as also observed by Peace et al. [18] and Steiger et al. [23]. Cultivar ‘344’ was grouped with ‘741’ and ‘660’, which is similar to previous observations made with RAF [18], isozyme [17], RAMiFi [24], and AFLP [23] markers. Population structure analysis revealed that cultivar ‘791’ constituted three or four distinct populations, which was supported by the observation of Peace et al. [18]. It was identified that ‘791’ is a tri-species cultivar containing *M*. *integrifolia*, *M*. *tetraphylla* and *M*. *ternifolia* in its ancestry. Our results additionally showed that ‘791’ may also have *M*. *jansenii* in its genetic background. We identified that ‘791’ shares almost equal amounts of *M*. *integrifolia* and *M*. *tetraphylla*. In contrast, Peace et al. [18] previously reported 10% *M*. *tetraphylla*, 55% *M*. *integrifolia* and 35% *M*. *ternifolia* in the ancestry of ‘791’. Interestingly, population structure analysis identified that two wild species *M*. *jansenii* and *M*. *ternifolia* represented the same population (Fig 4A). Although no historical relationships among these two wild species were evidenced, there are some clear morphological similarities [1]. For example, both *M*. *jansenii* and *M*. *ternifolia* are relatively dwarf accessions, produce pink red leaf flushes and flowers, and have bitter nuts. Previous isozyme analysis showed that *M*. *jansenii* and *M*. *ternifolia* are closely related [67], as observed in RAF [68] and STMS (sequence-tagged microsatellite site) studies [3], whilst Waldron et al. [68] and Peace et al. [3] suggested that *M*. *ternifolia* and *M*. *jansenii* were of sister species. An investigation on large numbers of wild accessions would be instrumental to explore differentiation among the four macadamia species. Identification of markers showing the signature of domestication could be the focus of future studies.

Though Australian elite hybrids were distributed across the four clusters, the ancestral relationship determined by both silicoDArT and SNP markers was consistent. Dwarf cultivars ‘DW1’, ‘DW2’, ‘DW3’, and ‘DW4’, and selections ‘QB-35-10’, ‘QB-5-81’, and ‘QB-7-11’ were grouped with their common parent ‘NG8’. The cultivar ‘762’ was also a common parent of ‘DW1’, DW2’, ‘QB-7-11’, and ‘QB-5-81’, and hence sub-grouped. Similarly, other elites ‘TF-9-22B’, ‘TF-44-15D’, ‘QB-9-72K’, and ‘QB-7-109L’ clustered with common parent ‘Daddow’. Consistency in determining pedigree relationships suggested that both DArT platforms are highly reliable for genetic diversity study in macadamia.

### DArTseq based SNP markers are useful tools for plant identity confirmation

The use of molecular markers for plant identity and parentage analysis has increased over the last two decades [69]. Although microsatellite markers were used previously in parentage analysis [25], this study was the first to use DArT markers in parentage analysis in macadamia. Using SNP markers, our study successfully predicted the plant identity and probable mother identities of plants in a rootstock trial. Most of the predictions validated field observations (S7 Table). DArTseq-based SNP markers are therefore useful to confirm plant identity through parentage analysis of macadamia progeny.

## Conclusions

Our study identified that both DArT platforms are of high quality, supported by the quality parameters and close relationships of the replicates of each cultivar in the neighbourhood join dendrograms. Both DArT markers successfully reflected the parental relationships and the extent of diversity in the population. Structure analysis clearly separated the population of various origins. DArTseq-based SNPs successfully demonstrated population structure across cultivars and confirmed plant identity in a mismatched rootstock trial. We therefore suggest that both silicoDArT and SNP markers are robust and an inexpensive option for breeders for genomics studies in macadamia. Both DArT platforms developed a large number of highly polymorphic markers, and hence are useful for linkage mapping in macadamia.

## Supporting information

S1 Table List of silicoDArT markers. (XLSX)

S2 Table List of SNP markers. (XLSX)

S3 Table Summary statistics of informative markers of both markers of DArT platform. (XLSX)

S4 Table Dissimilarity index among macadamia cultivars estimated by weighted neighbour-join analysis of silicoDArT markers in DARwin software. (XLSX)

S5 Table Dissimilarity index among macadamia cultivars estimated by weighted neighbour-join analysis of DArTseq-based SNP markers in DARwin software. (XLSX)

S6 Table Linear genetic distances across the cultivars and mismatched scions estimated from SNP markers using GenAlEx software. (XLSX)

S7 Table Dissimilarity results of plant identity and maternity analysis of phenotypically mismatched scions in a rootstock trial. (XLSX)
